# Electrochemically hydrogenated TiO_2_ nanotubes with improved photoelectrochemical water splitting performance

**DOI:** 10.1186/1556-276X-8-391

**Published:** 2013-09-18

**Authors:** Chen Xu, Ye Song, Linfeng Lu, Chuanwei Cheng, Dongfang Liu, Xiaohong Fang, Xiaoyuan Chen, Xufei Zhu, Dongdong Li

**Affiliations:** 1 Key Laboratory of Soft Chemistry and Functional Materials of Education Ministry, Nanjing University of Science and Technology, Nanjing 210094, China; 2 Shanghai Advanced Research Institute, Chinese Academy of Sciences, 99 Haike Road, Zhangjiang Hi-Tech Park, Pudong, Shanghai 201210, China; 3 Shanghai Key Laboratory of Special Artificial Microstructure Materials and Technology & School of Physics Science and Engineering, Tongji University, Shanghai 200092, China

**Keywords:** TiO_2_ nanotubes, Electrochemical reductive doping, Hydrogenation, Photoelectrochemical water splitting

## Abstract

One-dimensional anodic titanium oxide (ATO) nanotube arrays hold great potential as photoanode for photoelectrochemical (PEC) water splitting. In this work, we report a facile and eco-friendly electrochemical hydrogenation method to modify the electronic and PEC properties of ATO nanotube films. The hydrogenated ATO (ATO-H) electrodes present a significantly improved photocurrent of 0.65 mA/cm^2^ in comparison with that of pristine ATO nanotubes (0.29 mA/cm^2^) recorded under air mass 1.5 global illumination. The incident photon-to-current efficiency measurement suggests that the enhanced photocurrent of ATO-H nanotubes is mainly ascribed to the improved photoactivity in the UV region. We propose that the electrochemical hydrogenation induced surface oxygen vacancies contribute to the substantially enhanced electrical conductivity and photoactivity.

## Background

Continued research efforts over the past few decades on solar water splitting have led to a substantial improvement in both scientific understanding and technical application [1-4]. Because of its abundance, nontoxicity, and stability, TiO_2_ is one of the most promising photoanodes in the solar water splitting system. So far the solar-to-hydrogen (STH) efficiency of TiO_2_-based photoanodes is limited by a wide bandgap (3.0 to 3.2 eV) and numerous electron–hole recombination centers [5]. A variety of approaches have been explored to enhance the visible light activity of TiO_2_, such as metal doping [6] or nonmetal doping [7,8].

Recently, hydrogenation of TiO_2_, with intentionally introduced Ti^3+^ or oxygen vacancy states, has been proved to be an effective strategy for improving the electronic conductivity and photoresponse property [9-14]. Annealing processes in hydrogen atmosphere either under high temperature [13,14] or by a long processing duration [11] are two most employed ways. However, the need for either high-energy consumption or expensive facility would limit its practical application. Alternatively, the electrochemical reductive doping process provides another simpler approach for TiO_2_ hydrogenation. Under an external electric field, hydrogen is driven into the TiO_2_ lattice and reduces Ti^4+^ to Ti^3+^[15,16]. The intentionally introduced donor states associated with enhanced conductivity have delivered a variety of applications in template synthesis [17,18], electrochemical supercapacitors [19], and photovoltaic devices [20].

Moreover, in comparison with conventional nanoparticles, one-dimensional anodic titanium oxide (ATO) nanotube arrays with well-defined tubular structures provide a direct pathway for charge transport [21-23], thus possessing promising capabilities in photoelectrochemical (PEC) system. Herein, the electrochemical reductive doping approach is conducted on ATO nanotubes with the aim of improving the photoelectrochemical activity of TiO_2_ for hydrogen production through water splitting. The hydrogenated ATO nanotubes (ATO-H) showed significantly increased UV light response compared with the pristine ATO electrode. The hydrogen-induced oxygen vacancies in ATO-H are responsible for the improved conductivity and photoresponse.

## Methods

Ti foils (99.7%, 0.2 mm thickness, Shanghai Shangmu Technology Co. Ltd) were ultrasonically cleaned in acetone, ethanol, and deionized water successively after an annealing process (450°C for 2 h). Then electrochemical polish was carried out in a solution of acetic acid and perchloric acid which determined the flat surface of the Ti foils. ATO nanotube films were made by two-step anodization in ethylene glycol electrolyte containing 0.3 wt.% NH_4_F and 10 vol.% H_2_O. First-step anodization was performed at 150 V for 1 h in a conventional two-electrode configuration with a carbon rod as cathode electrode. The as-anodized nanotube films were removed from the Ti foil with adhesive tape [20]. Second-step anodization was performed under the same condition for 1 h. The ATO products were crystallized in ambient air at 150°C for 3 h, then up to 450°C for 5 h with a heating rate of 1°C/min.

The electrochemical reductive doping process was performed in a two-electrode system at room temperature, employing the ATO nanotubes and a Pt electrode as the cathode and anode, respectively, with the distance between the two electrodes exactly fixed at 2.5 cm. The crystallized ATO nanotubes were immersed in 0.5 M Na_2_SO_4_ aqueous solution, and a voltage of 5 V was imposed between the electrodes. The reductive doping duration was maintained in the range of 5 to 40 s, and the optimum time was found to be 10 s. Finally, the ATO nanotubes were taken out, washed with deionized water, and dried for measurements.

The morphology and crystalline structure of nanotube films were characterized using field-emission scanning electron microscope (FESEM, FEI Quanta 600, FEI Company, Hillsboro, OR, USA), transmission electron microscope (HRTEM, JEM-2100F, JEOL Ltd., Akishima, Tokyo, Japan), and X-ray diffractometer (XRD, D8 Discover diffractometer, Bruker AXS GMBH, Karlsruhe, Germany). Raman spectroscopy (DXR Raman microscope with 532-nm excitation laser, Thermo Fisher Scientific, Waltham, MA, USA) was employed for chemical state analysis. Time-resolved photoluminescence (TRPL) spectra were recorded at ambient temperature with a time-correlated single-photon counting (TCSPC) spectrometer (Photon Technology International, Inc., Birmingham, NJ, USA), where a pulsed laser at 375 nm with an average power of 1 mW (100 fs, 80 MHz) was used as the excitation source.

The PEC water splitting performances of the ATO nanotubes without and with electrochemical hydrogenation were evaluated by AUTOLAB using a three-electrode configuration with the nanotube films (1 × 1 cm^2^) as working electrode, Ag/AgCl (3 M KCl) electrode as reference electrode, and a platinum foil as counter electrode. The supporting electrolyte was 1 M potassium hydroxide (KOH, pH = 14) containing 1 wt.% of ethylene glycol solution, where ethylene glycol acted as a potential hole scavenger (electron donor) to minimize the recombination of charge carriers [24]. The photocurrent was measured at a potential of 0 V (vs Ag/AgCl) under chopped light irradiation with UV light (5.8 mW/cm^2^ at 365 nm) and simulated solar illumination (100 mW/cm^2^) from a Xe lamp coupled with an air mass 1.5 global (AM 1.5G) filter (Newport no. 94063A). The incident photon-to-current conversion efficiency (IPCE, DC mode) was measured in three-electrode configuration by an AUTOLAB electrochemical station with the assistance of a commercial spectral response system (QEX10, PV Measurements, Inc., Boulder, CO, USA). In order to record the stable photoresponse from photoanodes, each wavelength was held for 3 min before the photocurrent measurements. Impedance measurements were performed under dark condition at open-circuit potential over a frequency range of 100 kHz to 0.1 Hz with an amplitude of 10 mV.

## Results and discussion

**Figure 1:**
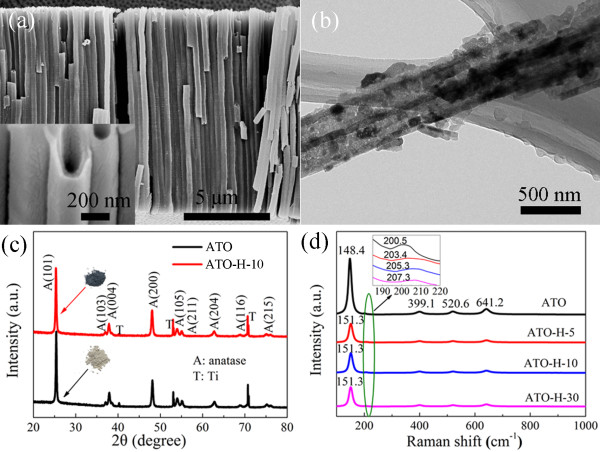
**The morphology and structure characterization of ATO and ATO-H. (a)** A side view of ATO nanotube film after second-step anodization. Inset of (a) shows an enlarged image indicating a smooth tube wall. **(b)** A TEM image of ATO nanotubes. **(c)** XRD patterns of pristine ATO and ATO-H-10 films. Inset of (c) shows the photographs of ATO and ATO-H nanotube powders. **(d)** Raman spectra of the pristine ATO and ATO-H nanotubes with different processing time (5, 10, and 30 s).

Figure 1a represents the cross-sectional views of ATO film after second-step anodization in which a vertically aligned one-dimensional feature is observed. The average outer diameter of nanotubes is approximately 300 nm, with a tube wall thickness around 75 nm. Figure 1b shows the TEM image of two adjacent nanotubes with crystalline nanoparticles (*D* = 25 to 50 nm) arranged along the nanotubes. The XRD patterns of the ATO and ATO-H nanotube films are shown in Figure 1c. Except for the peaks at 40.25°, 53.06°, and 70.71° that originated from the Ti metal, all other peaks are coincident with each other and can be indexed to anatase TiO_2_ (JCPDF no. 21–1272). The average crystallite size variation from 31.9 nm (ATO) to 31.3 nm (ATO-H), estimated from the major diffraction peak (2*θ* = 25.17°) using Scherrer's equation [25], is less than 2%. After scraping the ATO nanotube powders off the Ti foil substrates with a razor blade, a distinct color evolution is revealed from white (ATO powder) to blue-black (ATO-H-10) (inset of Figure 1c). The evolution of optical properties could be ascribed to the increased defect density [11] on tube surface as disclosed by the Raman spectroscopy analysis.

Figure 1d displays the Raman spectra of ATO nanotubes treated with different reductive processing times (denoted as ATO-H-5, ATO-H-10, and ATO-H-30 for 5-, 10-, and 30-s treatments, respectively). The six Raman vibrational mode of anatase TiO_2_ samples [26] can be found at 148.4 cm^-1^ (*E*_g_(1)), 200.5 cm^-1^ (*E*_g_(2)), 399.1 cm^-1^ (*B*_1g_(1)), 641.2 cm^-1^ (*E*_g_(3)), 520.6 cm^-1^ (*A*_1g_), and 519 cm^-1^ (*B*_1g_(2) superimposed with 520.6 cm^-1^), which is in agreement with the above XRD results. A slight blueshift and broadening of *E*_g_(1) and *E*_g_(2) peaks are observed in the ATO-H-10 sample, suggesting increased surface disorder due to the introduced oxygen vacancies [10]. According to the above analysis, the possibly introduced defect states originate from the formation of oxygen vacancies on ATO nanotubes.

**Figure 2:**
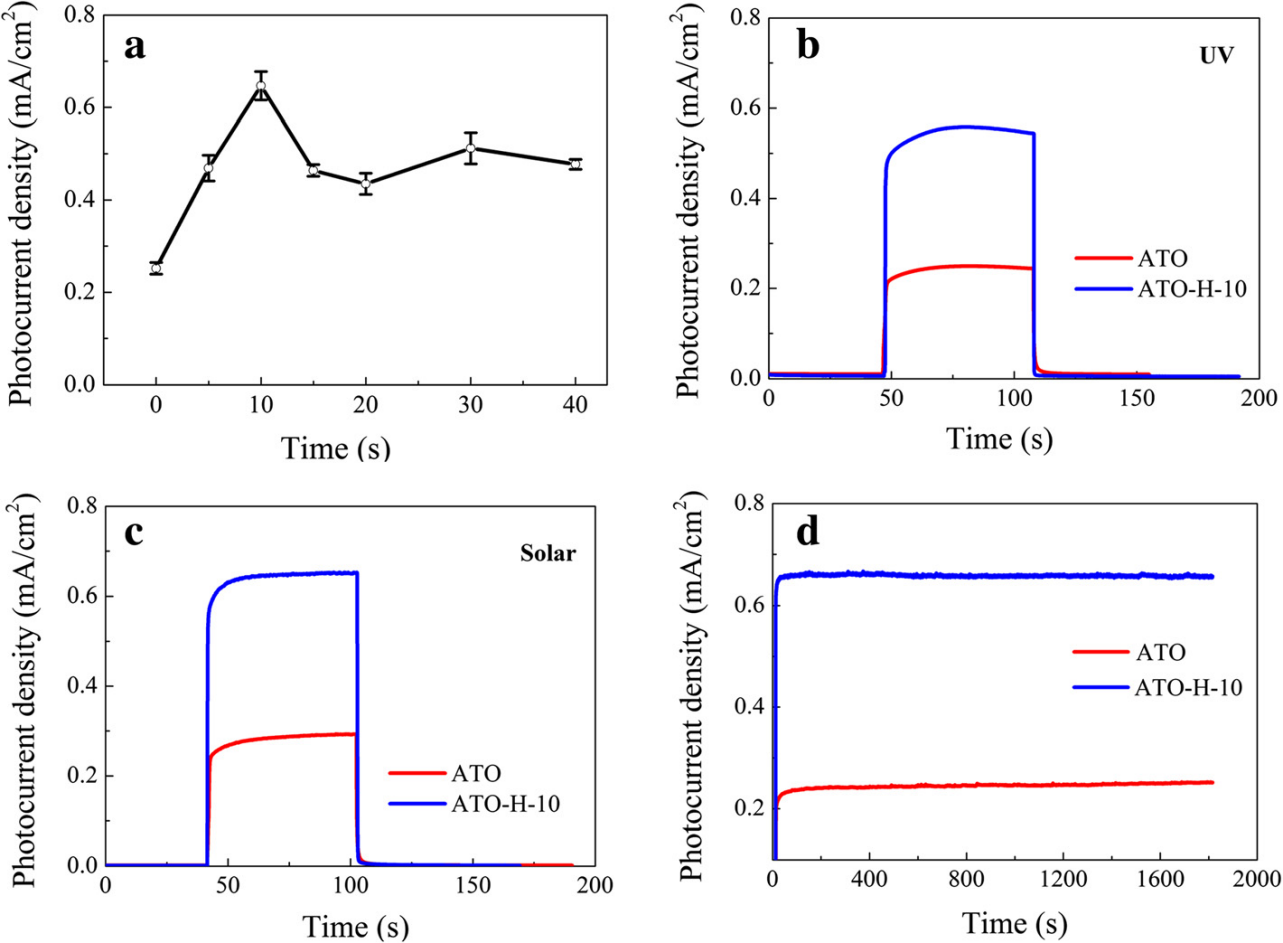
**Photocurrent responses of photoanodes at a constant bias of 0 V (vs Ag/AgCl). (a)** Photocurrent densities of ATO and ATO-H as a function of hydrogenation processing time. Photocurrent response of ATO and ATO-H-10 electrodes irradiated with **(b)** UV (365 nm) and **(c)** simulated solar light for 60 s light on. **(d)** Amperometric *I*-*t* curves of ATO and ATO-H-10 electrodes obtained under simulated solar illumination.

The photocurrent densities of ATO-H photoanodes at a constant potential of 0 V (vs Ag/AgCl) under the standard AM 1.5G solar light illumination are subsequently recorded as a function of reductive doping duration with respect to pristine ATO electrode (Figure 2a). Each duration is measured in at least three samples to average out the experimental fluctuation. The photocurrent densities increase gradually with the processing time, yielding a maximum value of 0.65 mA/cm^2^ for a 10-s treatment. Further prolonged processing time leads to a depressed performance, which could be ascribed to increased surface defect density and corresponding recombination rate. Thus, ATO-H electrodes with a 10-s doping duration (ATO-H-10) are employed in the following experiments unless otherwise specified.

Figure 2b, and c show the photocurrent of ATO and ATO-H-10 under illuminations of chopped UV (5.8 mW/cm^2^ at 365 nm) and simulated solar light (100 mW/cm^2^) at a constant potential of 0 V (vs Ag/AgCl). In comparison with the photocurrent density generated on pristine ATO (0.25 mA/cm^2^ under UV irradiation and 0.29 mA/cm^2^ under solar irradiation), the ATO-H-10 electrode delivers a much improved performance (0.56 mA under UV irradiation and 0.65 mA/cm^2^ under solar irradiation). Meanwhile, Figure 2d presents the chronoamperometric curves under simulated solar illumination for characterizing the long-term stability of nanotube photoelectrodes. Both curves were kept stable within the measurement period, indicating good stability after electrochemical hydrogenation.

Linear sweeps voltammetry (LSV) is a voltammetric method where the potential between the working electrode and a reference electrode is linearly swept in time with simultaneously recorded current. In the PEC water-splitting system, LSV is widely employed to characterize the photoelectrodes’ performance with quantitative open circuit voltage (*V*_oc_), short-circuit current (*J*_sc_), fill factor (FF), and light-to-hydrogen efficiency. However, unlike most solid-state solar cells, the linear sweeps in this liquid system are strongly dependent on the scan rate [27]. Under a fast potential scan, the thickness of diffusion layer will decrease from the electrode in comparison with the one under a slow scan. Consequently, the ionic flux towards electrode surface associated with current density will be increased. Therefore, the scan rate is worthy of serious consideration in evaluating the electrode performance. One could give an overestimated and misleading STH efficiency if an inappropriate high scan rate was applied.

**Figure 3:**
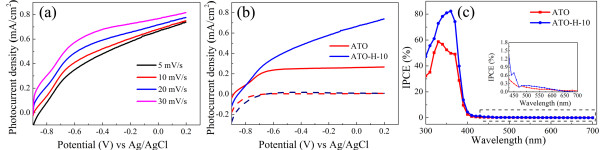
**PEC measurements on ATO and ATO-H-10. (a)** LSV curves of ATO-H-10 photoanode as a function of scan rates in 1 M KOH under simulated solar illumination. **(b)** LSV curves of pristine ATO and ATO-H-10 with a scan rate of 5 mV/s under simulated solar illumination. **(c)** IPCE spectra of pristine ATO and ATO-H-10 in the range of 300 to 700 nm at 0 V (vs Ag/AgCl). Inset: magnified IPCE spectra, highlighted in dashed box, at the incident wavelength range of 430 to 700 nm.

Figure 3a shows the LSV curves of ATO-H-10 measured as a function of scan rates. The photocurrent densities are elevated within the entire potential window by increasing the scan rate. A low scan rate of 5 mV/s is adapted in the following experiments, which will accommodate better with the results in photocurrent transients. Figure 3b shows the LSV characteristics of ATO and ATO-H-10 nanotubes under simulated solar illumination. The reductive doping process substantially improves the photocurrent density almost in the whole potential window except for a slightly decrease of *V*_oc_. The positive shift of *V*_oc_ indicates that the hydrogen-induced defects lead to a relatively faster recombination rate as proven by TRPL measurements (shown below). It is worth noting that the *J*_sc_ (0.66 mA/cm^2^) across the ATO-H-10 electrode (with a scan rate of 5 mV/s) is even higher than the highest photocurrent density (approximately 0.63 mA/cm^2^) ever reported on hydrogenated ATO nanotubes obtained from high-temperature annealing in hydrogen atmosphere (with a scan rate of 50 mV/s) [9].

The STH efficiency (*η*) on the photoanodes is calculated using the following equation [28]:

$$\eta =I\left(1.23-V\right)/J_{\text{light}}$$

where *V* is the applied bias voltage vs reversible hydrogen electrode (RHE), *I* is the photocurrent density at the measured bias, and *J*_light_ is the irradiance intensity of 100 mW/cm^2^. The pristine ATO exhibits a STH efficiency of 0.19% at -0.64 V (vs Ag/AgCl), while the ATO-H electrode yields a much improved efficiency (*η* = 0.30%) at -0.48 V (vs Ag/AgCl). Moreover, the quartz window reflects more than 4% of the solar irradiance [29], which means that the internal STH efficiencies are higher than the calculated values. Using front-side illumination configuration could reduce this loss and further boost the conversion efficiency [9].

IPCE measurements are carried out to investigate the contribution of each monochromatic light to the photocurrent density. Compared with the measurements based on the wide band light source without taking into account the differences between the spectra of the light source and the solar spectrum, and/or reliable calibration, which may vary from different research laboratories, the intensity-independent IPCE provides a reliable method to characterize the wavelength dependent photoresponse. The IPCE is calculated as a function of wavelength using IPCE = (1,240 (mW⋅nm/mA)*I*) / (*λJ*_light_), where λ is the incident light wavelength (nm) and *I* and *J*_light_ are the photocurrent density (mA/cm^2^) and incident light irradiance (mW/cm^2^) at a specific wavelength [28].

Figure 3c shows the IPCE plots of ATO and ATO-H-10 at zero bias vs Ag/AgCl. The results indicate that the enhanced photocurrent is mainly contributed by UV response due to electrical conductivity modification. Reductive doping gives rise to a pronounced enhancement in full UV region (300 to 400 nm) with a maximum value of 82% at 360 nm. The decrease at shorter wavelengths could be attributed to the unwanted light reflection or absorption before arriving to a photoanode [29]. In the longer wavelength region, IPCE plots represent abrupt decreases from approximately 49% (ATO) and approximately 74% (ATO-H-10) at 370 nm to less than 2% at 410 nm, which is determined by the recombination of charge carriers in the wide bandgap (approximately 3.2 eV) anatase TiO_2_[30]. A weak photoactivity of pristine ATO nanotube in 400 to 600 nm could be ascribed to fluorine doping during anodization in NH_4_F-containing electrolytes [9,31]. In addition, a slightly enhanced photocurrent can also be observed in the visible range (410 to 600 nm) on ATO-H-10 electrode (inset of Figure 3c). The oxygen vacancy states are generally localized with energies of 0.75 to 1.18 eV below the conduction band, which is lower than the redox potential for hydrogen evolution [32,33], while a high vacancy concentration could produce shallow donor levels just below the conduction band, which in turn provides enough energy for water splitting [34]. The experimental results suggest the formation of shallow levels which is responsible for the slightly enhanced visible light activity.

**Figure 4:**
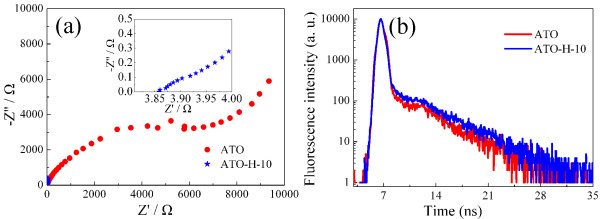
**Nyquist plots and TRPL spectra. (a)** Nyquist plots of electrochemical impedance spectra for ATO and ATO-H-10. **(b)** TRPL spectra of pristine ATO and ATO-H-10 films.

Further insight into the TiO_2_ characteristics is conducted by electrochemical impedance spectroscopy (EIS) measurements in the frequency range of 0.01 Hz to 100 kHz. Figure 4a shows the Nyquist plot of ATO and ATO-H-10 electrodes in dark condition. The intercepts of both plots on the real axis is less than 4 Ω, representing the conductivity of the electrolyte (*R*_s_). In contrast with the large semicircle diameter of pristine ATO electrode, an extremely small semicircle diameter for ATO-H-10 electrode (inset of Figure 4a) indicates a much improved electrode conductivity with significantly low charge transfer resistance [35].

It is known that PEC performance of the electrode is determined by charge separation and transfer process. Besides offering increased donor states, the introduced defect states would also serve as recombination centers for electron–hole pairs and consequently inhibit the charge collection. The visible luminescence band of anatase TiO_2_ is caused by donor-acceptor recombination, which is closely related to both trapped electrons and trapped holes [36]. In the nanocrystalline electrode, photoexcited carriers are readily captured in the inherent trap states. Trapping and thermally detrapping mechanisms will determine the slow decay process [37]. It is believed that the inherent shallow trap states in pristine ATO, serving as electron trapping sites, mainly contribute to the slow decay process. Subsequently, electrochemical hydrogenation could introduce more defect states into shallow energy levels to capture excited electrons, which will prolong the relaxation processes with the corresponding longer lifetime. The dynamic characteristics of photogenerated carriers are revealed by room-temperature TRPL spectroscopy. Figure 4b displays the TRPL curves of the different electrodes recorded at 413 nm with a 375-nm pulsed laser as excitation source. The ATO-H-10 electrode shows a somewhat longer lifetime compared with the pristine ATO electrode. This means that the electrochemical reductive process is an efficient strategy to improve electrode conductivity, with a slightly increased recombination rates. By utilizing single exponential decay fitting on the obtained curves, the averaged photoluminescence lifetimes of ATO and ATO-H-10 are calculated to be 537 and 618 ps, respectively.

## Conclusions

In conclusion, the electrochemical reductive doping processes are carried out to produce hydrogenated ATO photoanodes to improve PEC water splitting efficiency. A -5-V bias voltage, with only 10 s of processing time, yields a substantially enhanced photocurrent density of 0.29 to 0.65 mA/cm^2^. IPCE results indicate that the enhanced STH efficiency in ATO-H-10 is dominantly contributed by the improved photoactivities in the UV region. The electrochemically induced oxygen vacancies lead to increased donor density, which is responsible for the enhanced photocurrent with slightly increased parasitic recombination. This eco-friendly approach opens up a novel strategy for significantly improving the photoanode performance and provides potential for large-scale productions.

## Competing interests

The authors declare that they have no competing interests.

## Author’s contributions

XYC, XFZ, and DDL designed the experiments. CX, XHF, and LFL carried out the experiments. CX, YS, CWC, and DFL performed electrode characterization and data analysis. CX and DDL wrote the paper. All authors read and approved the final manuscript.
